# Parental Preconception Exposures to Outdoor Neighbourhood Environments and Adverse Birth Outcomes: A Protocol for a Scoping Review and Evidence Map

**DOI:** 10.3390/ijerph18178943

**Published:** 2021-08-25

**Authors:** Suzanne Mavoa, Daniel Keevers, Stefan C. Kane, Melissa Wake, Rachel Tham, Kate Lycett, Yen Ting Wong, Katherine Chong

**Affiliations:** 1 Melbourne School of Population & Global Health, University of Melbourne, Parkville, VIC 3010, Australia; 2 Murdoch Children’s Research Institute, Parkville, VIC 3052, Australia; melissa.wake@mcri.edu.au (M.W.); k.lycett@deakin.edu.au (K.L.); 3 Mary MacKillop Institute for Health Research, Australian Catholic University, Melbourne, VIC 3000, Australia; rachel.tham@acu.edu.au; 4 Melbourne Medical School, University of Melbourne, Parkville, VIC 3010, Australia; dkeevers@student.unimelb.edu.au; 5 Department of Obstetrics and Gynaecology, University of Melbourne, Parkville, VIC 3010, Australia; kane.s@unimelb.edu.au; 6 Department of Maternal Fetal Medicine, Royal Women’s Hospital, Parkville, VIC 3052, Australia; 7 Department of Paediatrics, University of Melbourne, Parkville, VIC 3010, Australia; 8 Liggins Institute, University of Auckland, Grafton, Auckland 1023, New Zealand; 9 School of Psychology, Faculty of Health, Deakin University, Burwood, VIC 3125, Australia; 10 IMPACT Institute, School of Medicine, Deakin University, Waurn Ponds, VIC 3216, Australia; yen.wong@deakin.edu.au; 11 Ingram School of Nursing, McGill University, Montreal, QC H3A 2M7, Canada; katherine.chong2@mail.mcgill.ca

**Keywords:** preconception, natural environment, built environment, pregnancy outcomes, birth outcomes, scoping review protocol

## Abstract

Parental preconception exposures to built and natural outdoor environments could influence pregnancy and birth outcomes either directly, or via a range of health-related behaviours and conditions. However, there is no existing review summarising the evidence linking natural and built characteristics, such as air and noise pollution, walkability, greenness with pregnancy and birth outcomes. Therefore, the planned scoping review aims to collate and map the published literature on parental preconception exposures to built and natural outdoor environments and adverse pregnancy and birth outcomes. We will search electronic databases (MEDLINE, EMBASE, Scopus) to identify studies for inclusion. Studies will be included if they empirically assess the relationship between maternal and paternal preconception exposures to physical natural and built environment features that occur outdoors in the residential neighbourhood and adverse pregnancy and birth outcomes. Two reviewers will independently screen titles and abstracts, and then the full text. Data extraction and assessment of study quality will be performed by one researcher and checked by a second researcher. Results will be summarised in a narrative synthesis, with additional summaries presented as tables and figures. The scoping review will be disseminated via a peer-reviewed publication, at academic conferences, and published on a website.

## 1. Introduction

### 1.1. Rationale

Adverse pregnancy and birth outcomes are an ongoing health challenge globally. They also have lifelong consequences, with small and preterm infants having an increased risk of hypertension [1], type 2 diabetes [1], cardiovascular disease [1,2], asthma [3], and poorer mental health [4] in later life. Similarly, congenital anomalies cause infant mortality and contribute to preterm birth, but also childhood morbidity [5]. For mothers, transient non-communicable diseases experienced during pregnancy, such as gestational diabetes and preeclampsia, present an emergent risk for later-life maternal non-communicable diseases [6,7,8]. Additionally, poor perinatal maternal mental health, while being an adverse outcome for the mother, also influences later child outcomes [9,10].

Built and natural outdoor environments are important, yet underappreciated, avenues to intervene to improve pregnancy and birth outcomes at the population level. Considerable evidence shows that environmental characteristics near the home (i.e., pollutants, natural environments, built environments including access to a range of facilities and services) are associated with health-related outcomes in both children and adults [11,12,13,14]. There is also accumulating evidence highlighting the importance of exposure to different types of outdoor environments during pregnancy. For instance, air pollution [15,16,17], environmental noise [18,19] and green space [20,21,22] have all been linked to adverse pregnancy and/or birth outcomes. However, far less attention has been directed at the role that these same environments play prior to conception. 

Preconception is a critical window of susceptibility [23,24,25]. Experimental animal studies and limited human studies show that conditions parents experience prior to conception can affect maternal and child health [25,26,27,28]. This is likely to occur through epigenetic mechanisms [29,30], which, given a single DNA sequence, result in different gene activity states [31]. Of interest here is the potential for epigenetic changes to occur as a result of environmental exposures, and through transgenerational inheritance [31]. While there is no agreed upon definition of preconception [32], previous research has considered preconception periods that range from months to years including prenatal or pubertal development [26,27,33]. While certain time windows are considered ‘critical’ or ‘sensitive’ it is important to note that preconception exposures may also have cumulative effects [26].

Outdoor built and natural environments therefore, could well play a role in preconception health. There is a small but growing body of evidence demonstrating that maternal preconception exposure to environmental toxins–including air pollutants that commonly occur in residential neighbourhoods (e.g., particulate matter, sulphur dioxide)-adversely affects pregnancy and birth outcomes [34,35]. There is even less evidence for fathers, despite animal studies demonstrating that paternal environmental exposures may affect offspring health via epigenetic modifications transmitted through sperm [36,37,38,39,40,41]. Few studies have explored non-occupational paternal exposures and offspring health in the general population [27,42], and it is unclear whether paternal preconception exposures are linked with adverse birth outcomes [24,42,43,44]. 

Beyond air pollutants, other aspects of the built and natural environment could also play an important role in preconception health. We know these outdoor environments are associated with health-related behaviours and conditions, such as physical activity, sedentary behaviour, diet, obesity, mental health, stress, smoking, and alcohol consumption. For instance, neighbourhoods with highly connected streets, high population density and a variety of destinations are associated with higher levels of physical activity and less sedentary behaviour [45] and obesity [45,46]. Tobacco and alcohol outlet density are associated with smoking and alcohol consumption behaviours [47,48], and there is mixed evidence that access to healthy/unhealthy food retail locations is linked to diet and obesity [49,50,51]. Greener neighbourhoods with more parks and vegetation are also linked to higher levels of physical activity [52,53] mental health and wellbeing [52,54], and lower levels of stress [52] and obesity [53]. There is also emerging evidence that other natural features of the environment, such as water and biodiversity may be linked to health [13,54,55,56,57]. Finally, there is some evidence that the psychosocial factors may also be linked to natural and built environments [58,59,60].

These same behaviours and conditions, which are in part determined by built and natural outdoor environments, also contribute to preconception health and to pregnancy and birth outcomes. For instance, the preconception diet for mothers predicts risk of gestational diabetes [61], preeclampsia [62] and preterm birth [63], with mixed evidence on the role of maternal diet on the child’s birth weight [63,64]. Psychosocial factors, such as personality traits, substance abuse, support, stressful life events are also known to be risk factors for maternal perinatal mental health [65]. Similarly, maternal preconception physical activity, mental health, obesity, smoking and alcohol consumption have all been linked to pregnancy and birth outcomes [28,61,66,67]. 

We know less about the impact of paternal preconception behaviours and health on birth outcomes [68]. Most evidence is based on animal models, and a few recent studies suggested that the paternal preconception environment, such as diet and early-life stress or trauma plays a role in foetal development and offspring health outcomes [69,70,71,72,73]. A handful of human studies have shown links between paternal alcohol consumption, smoking and mental health in the preconception period with birth outcomes [66,74,75], although in some cases the evidence is mixed [74]. Several studies have demonstrated that paternal health status is linked with preterm birth and low birth weight in the child, as well as gestational diabetes and preeclampsia in the mother [76,77]. However, there is likely substantial confounding factors since both parents often live at the same address and undertake activities together, resulting in similar environmental exposures. Thus, it is likely to be challenging to disentangle the impact of paternal and maternal preconceptional natural and built environment exposures, especially when considering shorter preconception periods. 

In summary, parental preconception exposures to built and natural outdoor environments could influence pregnancy and birth outcomes either directly, or via a range of health-related behaviours and conditions. A clear understanding of these relationships would enable us to identify modifiable environmental and behavioural risk factors that could improve the lifelong health of mothers, fathers, and infants. Yet the existing evidence is sparse and scattered, with a necessary first step being a coherent and systematic appraisal of what evidence exists and key evidence gaps. This is a task that is best suited to a systematic scoping review [78], which aims to provide an overview of the available evidence rather than a systematic evidence review, which focuses on a specific question and usually aims to determine causality, efficacy/effectiveness and/or effect size. Therefore, we have planned a scoping review to collate and map the published literature on parental preconception exposures to built and natural outdoor environments and adverse pregnancy and birth outcomes. 

### 1.2. Objectives

Since there are no existing scoping or systematic reviews on parental preconception exposures to outdoor natural and built environments, we will conduct a scoping review that aims to:

(1) Identify and characterise the existing scientific evidence on relationships between maternal and paternal outdoor residential neighbourhood environments in the preconception period and adverse pregnancy and birth outcomes.
(2) Identify evidence gaps and publish a narrative summary of the review and evidence map.

Previous versions of the protocol are registered in Zenodo. Version 1 was the initial protocol. We modified this prior to submission of this manuscript (Version 2). This manuscript represents the current version of the protocol. Any future modifications to this protocol will be similarly documented (including date and description of change), registered in Zenodo and reported in the final review paper.

## 2. Methods and Analysis

### 2.1. Information Sources and Search Strategy

We will identify relevant peer-reviewed published literature by searching the following databases: PubMed, Embase, CINAHL and Scopus. The search terms will cover three topics: (1) the outdoor neighbourhood environment, (2) the preconception period, and (3) adverse pregnancy and birth outcomes. The specific search strings for each database and the initial number of hits are provided in Table S1. The search will be restricted to literature published in the English language. The publication date will not be restricted.

### 2.2. Eligibility Criteria

Eligibility for inclusion is based on the Populations, Exposures, Comparators, and Outcomes PECO statement (Table 1). To be included in this systematic evidence map, studies must contain primary research investigating the relations between one or more of the specified outdoor environment exposures and one or more adverse birth outcomes.

Preconception exposures: Given the variation in definition of the preconception period [26,33], we will include all studies that explicitly state that they are investigating the preconception period regardless of how they define or measure this. We will also include studies that (a) explicitly assess environments during preconception (e.g., knowing the address at one month prior to conception, and sourcing pollution data for that month), and/or (b) make assumptions about location or environmental conditions during the preconception period (e.g., using the address at birth as a proxy for the preconception address and sourcing average pollution data for the year of birth). 

Objectively assessed physical outdoor environment features: We will restrict exposures to the physical characteristics of the outdoor environment. These will include environmental pollutants most commonly associated with residential neighbourhoods (e.g., traffic related air pollution, woodfire smoke, noise), weather/climate, and built and natural environmental features. We will focus only on the residential neighbourhood. We will include multiple definitions of residential neighbourhoods (administrative units, radial buffers, road network buffers) at any scale.

Additional inclusion criteria are:

- Objective and quantitative measurement of the environment.
- Peer reviewed, full text publications in the English language (acknowledging that this may result in English language bias).

### 2.3. Data Management and Selection Process

Search results will be imported into Covidence where the screening/selection process will be managed [79]. Duplicate records will be automatically removed during the import. First, two screeners will independently assess each title and abstract for relevance, with included literature moving to the next stage. Next, two screeners will assess the full text to determine whether the manuscript meets the inclusion criteria. Disagreements will be resolved by consensus, and if necessary, a third screener.

The number of studies evaluated at each step will be recorded. Any modifications to the search or protocol will be included as amendments to the registered protocol.

### 2.4. Data Extraction and Coding Strategy

Data shown in Table 2 will be extracted into a standardised Microsoft Excel form. Data will be collected at either the study or exposure-outcome level depending on the variable. The form allows for collection of the variables listed in Table 2. Two researchers (RT, KC) have already independently piloted the form for three studies. Any amendments to the form will be recorded in the registered protocol. 

Two researchers will independently extract the data from the included full text studies into the form. Inconsistencies will be discussed and if no agreement is reached, a third reviewer will decide. 

### 2.5. Quality Assessment

During data extraction, study quality will be assessed for each included study using the relevant Joanna Briggs Institute critical appraisal checklist [80,81]. 

### 2.6. Synthesis and Visualisation of Results

As is appropriate for a scoping review, we will systematically map the existing evidence on parental preconception exposures to the natural and built environment, and we will visualise the evidence using charts, tables and maps. We will also provide a narrative summary of the results. Gaps and trends in the evidence will be discussed with reference to study quality. The synthesis will be grouped by exposures, outcomes, and study regions.

## 3. Conclusions

This will be the first synthesis of evidence on parental preconception exposures to the broad range of natural and built environment features that we are exposed to in the course of our everyday lives. The findings from this review will be an important step towards helping to identify modifiable environmental and behavioural risk factors that could improve pregnancy and birth outcomes, as well as the lifelong health of mothers, fathers and infants. It will aid researchers by identifying key evidence gaps and important targets for future research and be of interest to stakeholders involved in urban environmental planning and design. The results will also be of practical use to primary care practitioners, who may use the findings to enhance evidence-based preconception patient education. Furthermore, these results will be applicable to clinicians’ assessments of women during the preconception period, as they may be better equipped to conduct an evidence-informed patient assessment related to environmental determinants of health. The review will be submitted for publication in a peer-reviewed journal and result summaries (e.g., the evidence map) will be published online. The scoping review does not require ethics approval since it consists of collecting and review publicly available documents.

## Figures and Tables

**Table 1:** Populations, Exposures, Comparators, and Outcomes (PECO) Statement.

Population	Pregnant and/or Postpartum Human Women of any Reproductive Age and Neonates.
Exposures	At least one feature of the physical outdoor residential neighbourhood environment that has been objectively assessed for the preconceptual period for one or both parents. Environmental features include pollutants (e.g., sulphur dioxide, nitrogen oxides, particulate matter), weather (e.g., temperature, humidity), built environment (e.g., dwelling density, walkability), and the natural environment (e.g., vegetation, coastline). The exposures may be individual exposures or aggregate indices. The preconceptual period includes the period prior to conception and during pregnancy (all-trimesters).
Comparators	(1) Mothers and (2) Fathers exposed to higher versus lower levels of the exposures.
Outcomes	At least one adverse pregnancy or birth outcome including: Maternal (during all trimesters of pregnancy and at/immediately following birth): gestational diabetes mellitus, gestational hyperglycaemia, pre-eclampsia, eclampsia, gestational hypertension, pregnancy complications, mental health, Neonate (assessed at/immediately following birth): low or large birth weight, gestational age, preterm birth, premature rupture of membranes, congenital anomalies, birth complications, stillbirths.

**Table 2:** Data extraction and coding variables. Import (I) indicates variable data will be imported from Covidence. Export (E) indicates the researcher will extract the data from the paper. Derive (D) indicates that the researcher will calculate the variable based on extracted data.

Variable	Import/Extract/Derive	Categories
Bibliographic information		
- authors	I	free text
- publication year	I	free text
- title	I	free text
- journal	I	free text
- citation	I	free text
Study information		
- study type	E	observational, natural experiment, intervention
- year/s conducted	E	free text
- number of mothers	E	free text (integer)
- number of fathers	E	free text (integer)
Study location		
- city/area/region	E	free text
- country	E	free text
- region	D	Africa, North America, Central/South America, Central Asia, East Asia, South East Asia, West Asia, Europe, Australasia, Other Oceania
- country income group	D	World Bank income group at first year of study: low, lower-middle, upper-middle, high
- urban/rural	E/D	urban, rural, both, unknown
Exposure information		
- paternal/maternal	E	maternal, paternal
- preconception definition	E	free text
- location used to represent residence	E	mother’s home, birth hospital, other
- residential location resolution	E	address, street, area unit, other
- timing of location details	E	preconception, pregnancy, birth, other
- neighbourhood definition	E	administrative unit, radial buffer, network buffer, other
- neighbourhood scale	E	free text (either a distance or the name of the administrative unit)
- outdoor environment feature	E	free text
- outdoor environment feature data source	E	free text
- time period of data source	E	free text
- exposure measurement	E	free text
Outcome information		
- timing of outcome assessment	E	free text
- preeclampsia	E	yes/no
- gestational diabetes	E	yes/no
- preterm birth	E	yes/no
- low birth weight	E	yes/no
- other outcome	E	free text
- preeclampsia measurement method	E	free text
- gestational diabetes measurement method	E	free text
- preterm birth measurement method	E	free text
- low birth weight measurement method	E	free text
- other outcome measurement method	E	free text
Covariate information		
- covariates/confounders	E	free text
Results		
- summary of results	E	free text
Critical appraisal checklist		

## Data Availability

Not applicable.

## Supplementary Materials

The following are available online at https://www.mdpi.com/article/10.3390/ijerph18178943/s1, Table S1: Search strings and number of hits for each database (searches conducted on 22 August 2021).
