# Supplementary material for: Parental Preconception Exposures to Outdoor Neighbourhood Environments and Adverse Birth Outcomes: A Protocol for a Scoping Review and Evidence Map
Source: Int J Environ Res Public Health. 2021 Aug 25;18(17):8943. doi: 10.3390/ijerph18178943 (PMC8431720; doi:10.3390/ijerph18178943)
Supplement: Supplementary file 1 [file ijerph-18-08943-s001.zip › ijerph-1339104-supplementary.pdf]

Parental preconception exposures to outdoor neighbourhood environments and adverse birth outcomes: a protocol for a scoping review and evidence map

Suzanne Mavoa[1,2,3] suzanne.mavoa@unimelb.edu.au
Daniel Keevers[4] dkeevers@student.unimelb.edu.au
Stefan C. Kane[5,6] kane.s@unimelb.edu.au
Melissa Wake[2,7,8] melissa.wake@mcri.edu.au
Rachel Tham[3] rachel.tham@acu.edu.au
Kate Lycett[2,7,9] k.lycett@deakin.edu.au
Yen Ting Wong[10] yen.wong@deakin.edu.au
Katherine Chong[11] katherine.chong2@mail.mcgill.ca

Supplementary Table S1. Search strings and number of hits for each database (searches conducted on 22 August 2021).

	Search string				Number of hits			
Search #	MEDLINE	Embase	Scopus	CINAHL	MEDLINE	Embase	Scopus	CINAHL
1	pre-pregnancy or prepregnancy or pre concept* or preconcept* or pre-gravid or pregravid or "before pregnancy" or "prior to pregnancy"	pre-pregnancy or prepregnancy or pre concept* or preconcept* or pre-gravid or pregravid or "before pregnancy" or "prior to pregnancy"	TITLE-ABS-KEY(pre-pregnancy or prepregnancy or pre concept* or preconcept* or pre-gravid or pregravid or "before pregnancy" or "prior to pregnancy")	pre-pregnancy or prepregnancy or pre concept# or preconcept# or pre-gravid or pregravid or "before pregnancy" or "prior to pregnancy"	21,207	31,682	68,030	1,224
2	generational or intergeneration	generational or intergeneration	TITLE-ABS-KEY(generational or	generational or intergenerational or	16,529	16,351	46,061	1,281

	al or cross-generational or transgenerational*	al or cross-generational or transgenerational*	intergenerational or cross-generational or transgenerational*)	cross-generational or transgenerational#				
3	neighborhood or neighbourhood or environment or environment* or exposure	neighborhood or neighbourhood or environment or environment* or exposure	TITLE-ABS-KEY(neighborhood or neighbourhood or environment or environment* or exposure)	neighborhood or neighbourhood or environment or environment# or exposure	2,154,317	2,827,823	6,566,439	51,763
4	(1 or 2) and 3	(1 or 2) and 3	(#1 or #2) and #3	(S1 or S2) and S3	8,641	10,754	20,161	351
5	"fetal growth restriction" or "foetal growth restriction" or FGR or "low birthweight" or "low birth weight" or LBW or "small for gestational age" or SGA or "intrauterine growth restriction" or IUGR or "intrauterine growth retardation" or "gestational age" or	"fetal growth restriction" or "foetal growth restriction" or FGR or "low birthweight" or "low birth weight" or LBW or "small for gestational age" or SGA or "intrauterine growth restriction" or IUGR or "intrauterine growth retardation" or "gestational age" or	TITLE-ABS-KEY("fetal growth restriction" or "foetal growth restriction" or FGR or "low birthweight" or "low birth weight" or LBW or "small for gestational age" or SGA or "intrauterine growth restriction" or IUGR or "intrauterine growth retardation" or "gestational age" or "fetal growth retardation" or "large for gestational age" or LGA or "large for dates" or	"fetal growth restriction" or "foetal growth restriction" or FGR or "low birthweight" or "low birth weight" or LBW or "small for gestational age" or SGA or "intrauterine growth restriction" or IUGR or "intrauterine growth retardation" or "gestational age" or "fetal growth retardation" or "large for gestational age" or LGA or "large for dates" or macrosomia or macrosomic or dystocia<br><br>OR MH Gestational age	193,163	281,393	275,754	52,944

	Gestational Age/ or "fetal growth retardation" or "large for gestational age" or LGA or "large for dates" or macrosomia or macrosomic or dystocia	Gestational Age/ or "fetal growth retardation" or "large for gestational age" or LGA or "large for dates" or macrosomia or macrosomic or dystocia	macrosomia or macrosomic or dystocia)					
6	preterm or prematur* or Fetal Membranes/ or Premature Rupture/ or PROM or "premature rupture"	preterm or prematur* or Fetal Membranes/ or Premature Rupture/ or PROM or "premature rupture"	TITLE-ABS-KEY(preterm or prematur* or "fetal Membranes" or PROM or "premature rupture")	preterm or prematur* or or or PROM or "premature rupture"<br><br>OR MH Fetal Membranes<br><br>OR MH Premature Rupture	256,003	363,293	360,666	35,808
7	preeclampsia or pre-eclampsia or "pre eclampsia" or eclampsia or "pregnancy induced hypertension" or "gestational hypertension"	preeclampsia or pre-eclampsia or "pre eclampsia" or eclampsia or "pregnancy induced hypertension" or "gestational hypertension"	TITLE-ABS-KEY(preeclampsia or pre-eclampsia or "pre eclampsia" or eclampsia or "pregnancy induced hypertension" or "gestational hypertension" or "hypertensive disorder of	preeclampsia or pre-eclampsia or "pre eclampsia" or eclampsia or "pregnancy induced hypertension" or "gestational hypertension" or "hypertensive disorder of pregnancy" or toxaemia or toxemia	55,3897	91,317	76,677	14,141

	or "hypertensive disorder of pregnancy" or toxaemia or toxemia	or "hypertensive disorder of pregnancy" or toxaemia or toxemia	pregnancy" or toxaemia or toxemia)					
8	"gestational diabetes" or GDM or "pregnancy induced diabetes" or "gestational hyperglycaemia" or "gestational hyperglycemia"	"gestational diabetes" or GDM or "pregnancy induced diabetes" or "gestational hyperglycaemia" or "gestational hyperglycemia"	TITLE-ABS-KEY("gestational diabetes" or GDM or "pregnancy induced diabetes" or "gestational hyperglycaemia" or "gestational hyperglycemia")	"gestational diabetes" or GDM or "pregnancy induced diabetes" or "gestational hyperglycaemia" or "gestational hyperglycemia"	17,778	28,603	23,245	8,374
9	(pregnancy or birth) adj (complication* or outcome* or defect*) or Pregnancy Complications/ or Infant, Newborn/ or Respiratory Therapy/	(pregnancy or birth) adj (complication* or outcome* or defect*) or Pregnancy Complications/ or Infant, Newborn/ or Respiratory Therapy/	TITLE-ABS-KEY((pregnancy or birth) pre/1 (complication* or outcome* or defect*) or "respiratory therapy")	(pregnancy or birth) adj (complication* or outcome* or defect*)<br><br>OR MH Pregnancy Complications<br><br>OR MH Infant, Newborn<br><br>OR MH Respiratory Therapy	807,117	741,772	245,282	159,643
10	((congenital or birth or fetal) adj (abnormal*	((congenital or birth or fetal) adj (abnormal*	TITLE-ABS-KEY(((congenital or birth or fetal) pre/1	((congenital or birth or fetal) adj (abnormal* or anomal* or defect* or	164,374	596,569	578,521	6,948

	or anomal* or defect* or disorder* or malform*)) or teratogenesis or Teratogens/ or Teratology/ or Mutagenesis/ or stillbirth or "fetal death" or "foetal death"	or anomal* or defect* or disorder* or malform*)) or teratogenesis or Teratogens/ or Teratology/ or Mutagenesis/ or stillbirth or "fetal death" or "foetal death"	(abnormal* or anomal* or defect* or disorder* or malform*)) or teratogen* or teratology or mutagenesis or stillbirth or "fetal death" or "foetal death")	disorder* or malform*)) or teratogenesis or stillbirth or "fetal death" or "foetal death"<br><br>OR MH Teratogens<br><br>OR MH Teratology<br><br>OR MH Mutagenesis				
11	((*natal or *partum or maternal) adj (depress* or anxiety or mental)) or Depression, Postpartum/	((*natal or *partum or maternal) adj (depress* or anxiety or mental)) or Depression, Postpartum/	TITLE-ABS-KEY(((*natal or *partum or maternal) pre/1 (depress* or anxiety or mental)))	((*natal or *partum or maternal) adj (depress* or anxiety or mental))<br><br>OR MH Depression, Postpartum	11,652	11,457	20,228	6,309
12	5 or 6 or 7 or 8 or 9 or 10 or 11	5 or 6 or 7 or 8 or 9 or 10 or 11	#5 or #6 or #7 or #8 or #9 or #10 or #11	S5 or S6 or S7 or S8 or S9 or S10 or S11	1,157,339	1,657,385	1,277464	220,443
13	"air pollut*" or "atmospheric pollut*" or noise or Traffic-Related Pollution/ or Air Pollution/ or Noise/	"air pollut*" or "atmospheric pollut*" or noise or Traffic-Related Pollution/ or Air Pollution/ or Noise/	TITLE-ABS-KEY("air pollut*" OR "atmospheric pollut*" OR traffic W/2 pollut* OR noise)	"air pollut*" or "atmospheric pollut*" or noise<br><br>OR MH Traffic-Related Pollution<br><br>OR MH Air Pollution	269,595	328,750	291,588	32,311

				OR MH Noise				
14	heat or temperature* or weather or climate or humidity or "light at night" or ALAN	heat or temperature* or weather or climate or humidity or "light at night" or ALAN	TITLE-ABS-KEY(heat or temperature* or weather or climate or humidity or "light at night" or ALAN)	heat or temperature* or weather or climate or humidity or "light at night" or ALAN	1,199,668	1,352,469	6,992,80 4	60,698
15	density or road or street or walkability or transit or "public transport" or "land use" or access* or "urban design" or "built form" or "built environment"	density or road or street or walkability or transit or "public transport" or "land use" or access* or "urban design" or "built form" or "built environment"	TITLE-ABS-KEY(density or road or street or walkability or transit or "public transport" or "land use" or access* or "urban design" or "built form" or "built environment")	density or road or street or walkability or transit or "public transport" or "land use" or access* or "urban design" or "built form" or "built environment"	1,471,286	2,025,104	5,566483	264,42 5
16	green space* or greenspace* or park* or open space* or forest* or canopy or tree* or greenness or vegetation or biodiversity or species or water or coast	green space* or greenspace* or park* or open space* or forest* or canopy or tree* or greenness or vegetation or biodiversity or species or water or coast	TITLE-ABS-KEY("green space*" or greenspace* or park* or "open space*" or forest* or canopy or tree* or greenness or vegetation or biodiversity or species or water or coast or sea or lake* or river* or	green space* or greenspace* or park* or open space* or forest* or canopy or tree* or greenness or vegetation or biodiversity or species or water or coast or sea or lake* or river* or bluespace* or blue space*	2,619,590	3,134,392	8,736,85 7	150,82 1

	or sea or lake* or river* or bluespace* or blue space*	or sea or lake* or river* or bluespace* or blue space*	bluespace* or "blue space*")					
17	13 or 14 or 15 or 16	13 or 14 or 15 or 16	#12 or #13 or #14 or #15 or #16	S13 or S14 or S15 or S16	4,953,915	6,120,555	19,558,280	486,008
18	12 and 17	12 and 17	#12 and #17	S12 and S17	93,431	149,342	1,277,464	12,755
**19**	**4 and 18**	**4 and 18**	**#4 and #18**	**S4 and S18**	**329**	**504**	**1,629**	**10**
